# Knowledge mapping and research trends on rehabilitation of patients with mechanical ventilation in the ICU from 2005 to 2024: a bibliometric analysis via CiteSpace and Bibliometrix

**DOI:** 10.3389/fneur.2025.1609558

**Published:** 2025-12-03

**Authors:** Xizhen Kang, Jun Tian, Yanan Yang, Ye Zhu, Hui Teng, Huifang Xiao, Qing Shu

**Affiliations:** 1 Department of Rehabilitation Medicine, Zhongnan Hospital of Wuhan University, Wuhan, China; 2 Department of Traditional Chinese Medicine, China Resources & Wugang General Hospital, Wuhan, China

**Keywords:** rehabilitation, mechanical ventilation, bibliometric analysis, intensive care unit, CiteSpace, Bibliometrix, Web of Science

## Abstract

**Objective:**

Early rehabilitation, which refers to multidisciplinary, structured interventions initiated during the acute phase of illness, aimed at promoting physical recovery, reducing disability, and preventing complications, is essential for critically ill patients with mechanical ventilation. To review literature related to early rehabilitation in patients with mechanical ventilation in the intensive care unit, the paper aims to identify research topics and frontiers, report on current research trends, and offer valuable insights and perspectives for future development in the field.

**Methods:**

This study retrieved related publications from the Web of Science Core Collection database on March 12, 2025. After collecting the data, CiteSpace V.6.1. R6 was used to conduct a visual analysis of countries, institutions, authors, cited journals, cited references, and keywords. Bibliometrix 4.1.3 was used to generate the main information, country collaboration map, and three-field plot. The data was visualized through knowledge maps and collaborative networks.

**Results:**

We obtained a total of 375 articles on early rehabilitation in patients with mechanical ventilation. The number of annual publications has generally shown a steady growth trend in the past 20 years, with an annual growth rate of 21.59%. The United States, Brazil, Australia, and England are major contributing countries. North American and European countries have established the most intensive cooperation networks. Most of the active scholars, institutions, and journals in this field come from the United States, Canada, Australia, and England. Our research shows that ICU-acquired weakness, pulmonary dysfunction, and disorder of consciousness are important issues as well as challenges that need to be addressed urgently.

**Conclusion:**

This study analyzed the current status of early rehabilitation in patients with mechanical ventilation in the intensive care unit via CiteSpace and Bibliometrix, then identified the research hotspots and frontiers on it. While current evidence remains limited, methodologically rigorous multicenter randomized controlled trials with large cohorts are warranted to establish robust evidence regarding rehabilitation efficacy in mechanically ventilated patients. Emerging innovations in rehabilitation protocols are anticipated to progressively optimize clinical pathways as research methodologies advance.

## 1 Introduction

Mechanical ventilation (MV) is a kind of life-support technique that is commonly used in the intensive care unit (ICU) to treat patients with life-threatening conditions (1). It can help patients maintain airway patency by means of ventilators, effectively prevent the body from hypoxia, and avoid respiratory failure. Studies show that up to 70% of ICU patients require MV during their ICU hospitalization (2, 3). However, during MV, patients are susceptible to a range of complications due to various factors. Firstly, immobility-related complications such as muscle atrophy, pressure ulcers, ventilator-associated pneumonia, and impaired physical function are common. Secondly, bedrest exerts negative impacts on multiple systems, including the respiratory, musculoskeletal, cardiovascular, nervous, and immune systems, leading to serious adverse outcomes. Finally, ICU-acquired weakness or delirium usually persists for months to years after discharge and significantly affects their quality of life (4–6). The mortality rate is 37% in adult patients undergoing MV, 17% in pediatric patients, and significantly higher in geriatric patients (7). According to statistical data, the daily cost associated with MV and ICU care has risen by 25.8%, imposing a significant economic burden on families and society (8). Given the high rates of complications, mortality, and economic burden associated with MV, early rehabilitation is essential and significant.

Early rehabilitation refers to multidisciplinary, structured rehabilitation interventions initiated during the acute or critical phase of an illness or injury, with a crucial application in the ICU, aimed at promoting physical recovery, reducing disability, and preventing complications (9, 10). Empirical evidence has consistently demonstrated that early rehabilitation is safe and effective for ICU patients receiving MV (11–13). The American Thoracic Society and the American College of Chest Physicians recommend that adults who require MV for more than 24 h during acute hospitalization should receive protocolized rehabilitation directed toward early mobilization (5). Nationwide surveys in Germany demonstrate that integrating prolonged weaning from MV with early rehabilitation significantly improves weaning success rates in critically ill patients (14, 15). Schreiber et al. retrospectively analyzed 1,313 patients who underwent 4-step intensive physical therapy and found that patients completing >2 steps had a significantly higher weaning success rate (16). Additionally, specific inspiratory muscle training has been shown to increase inspiratory muscle strength and improve maximum inspiratory pressure in ventilator-dependent ICU patients with MV (13). Early physical and occupational therapy can enhance patients’ functional independence, reduce the incidence of delirium and ICU-acquired weakness, and improve cognitive and psychological outcomes (17). The study also found that early rehabilitation was associated with independent activities of daily living at discharge (18). In fact, early rehabilitation interventions for critically ill patients with MV have evolved over the past two decades, during which numerous studies have shifted the treatment paradigm from “strict bedrest” to “limited activity” to “early mobilization” (19). This progression highlights the growing importance and momentum of early rehabilitation in the ICU (20). However, the research hotspots and trends in this field have not been systematically summarized.

Bibliometrics is a discipline that employs statistical and mathematical methods to quantitatively and qualitatively analyze the output and impact of publications within a specific research field (21). By analyzing information such as countries, institutions, keywords, etc., it can reveal connectivity density, collaboration patterns, contribution strength, and emerging trends within and across relevant research fields. To fill this knowledge gap, this study aimed to conduct a bibliometric analysis of early rehabilitation of patients with MV in the ICU over the past two decades (2005–2024) aiming to: (i) Construct a knowledge map of the field, including contributions from countries, institutions, authors, and journals; (ii) Identify research hotspots through keyword co-occurrence and clustering analysis; and (iii) Explore potential breakthroughs and future research trends in this field.

## 2 Materials and methods

### 2.1 Data source

Web of Science (WoS) is a leading global citation database renowned for its coverage of natural sciences, social sciences, arts, and humanities (22). Evaluations by Gusenbauer highlight WoS’s strengths in interdisciplinary coverage, citation tracking, and deduplication, significantly enhancing the accuracy and efficiency of bibliometric analysis (23, 24). Moreover, publications in the WoS Core Collection (WoSCC) are globally recognized for their high quality and impact (25). Therefore, WoSCC was selected as the sole data source for this study.

### 2.2 Search strategy

All the data was obtained from WoSCC on March 12, 2025. The data retrieval strategy was as follows: (i) Topic 1 = (“intensive care*” OR “ICU” OR “critical care*” OR “critically ill patients” OR “critical illness”) AND Topic 2 = (“rehabilitation” OR “physiotherapy” OR “physical therap*” OR “physical medicine” OR “early mobili*” OR “early ambulation” OR “exercise” OR “occupational therap*” OR “speech therap*” OR “swallowing therap*” OR “cognitive training” OR “psychological therap*” OR “muscle * training” OR “electrical stimulation*” OR “wake promot*” OR “promot* arousal” OR “arousal promot*” OR “wakefulness promot*” OR “promot* wakefulness”) AND Topic 3 = (“mechanical ventilat*” OR “invasive ventilation” OR “non-invasive ventilation”); (ii) Document Type = article; (iii) Language = English; (iv) Publication Year = 2005–2024.

### 2.3 Selection criteria

Inclusion criteria were: (1) The study population was clearly defined as ICU patients receiving MV (including invasive and non-invasive); (2) The research content must focus on the rehabilitation interventions for patients with MV, including but not limited to early mobilization, physical therapy, occupational therapy, cognitive training, and other related approaches; (3) Original articles (e.g., randomized controlled trial, cohort study); (4). Articles published from 2005 to 2024; (5) Only studies focusing on adult patients were included.

Exclusion criteria were: (1) Publications not in English; (2) Animal or *in vitro* experiments; (3) Not-original research (e.g., review, editorial, meeting); (4) Duplicate publication, only the latest or most complete version was retained; (5) Retracted publications.

**Figure 1:**
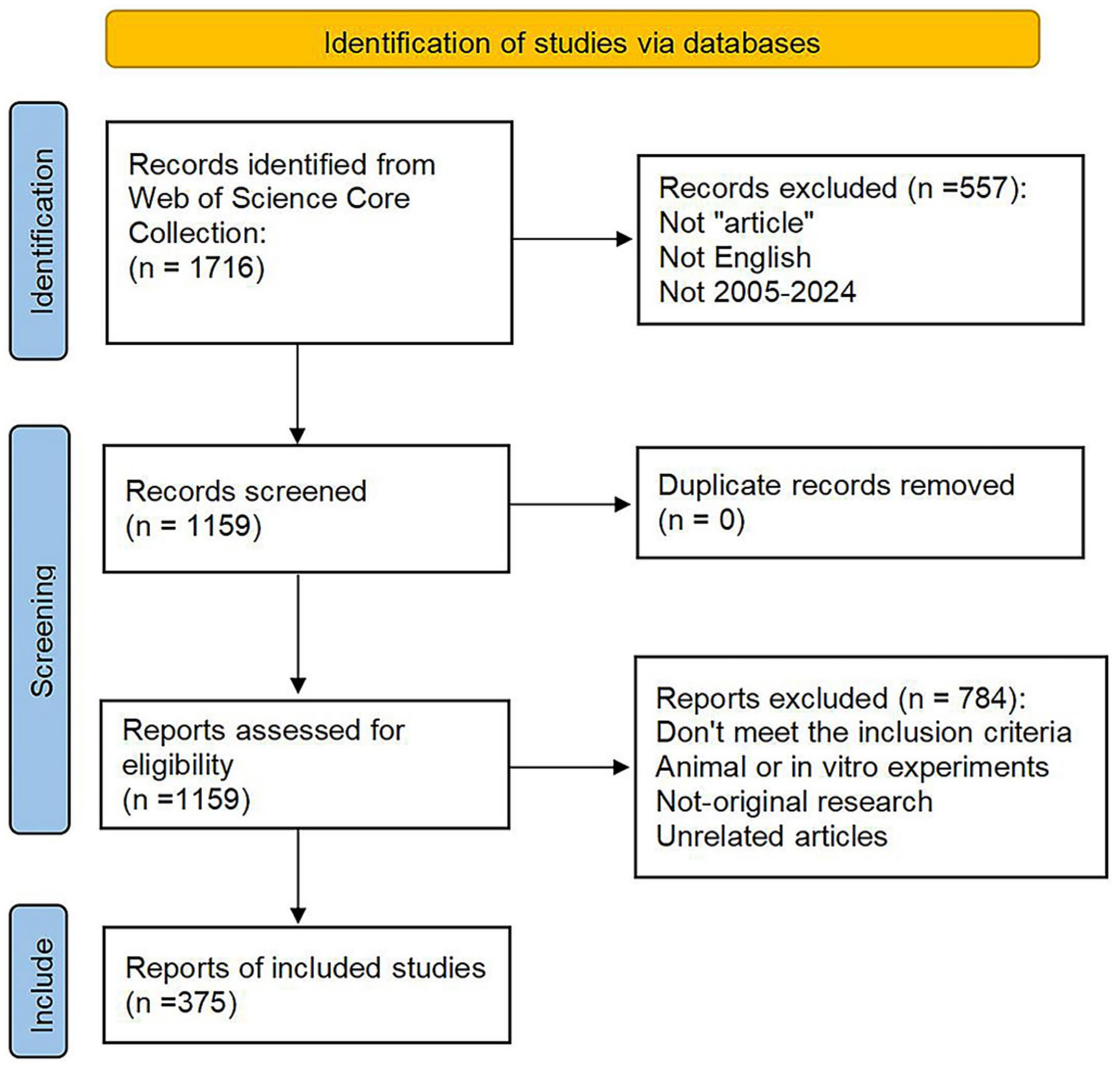
The flowchart of literature screening in this study.

Literature screening was conducted by at least two independent reviewers. One reviewer had a doctoral degree and rich clinical rehabilitation expertise, and another had a master’s degree and extensive experience in literature review. Discrepancies were resolved through discussion or, if necessary, by consulting a third expert. This approach ensures the objectivity and accuracy of the study selection. Finally, full records and their corresponding cited references were downloaded in plain text format, and the exported document was named “download_XXX.txt.” And then imported into the CiteSpace and Bibliometrix for visual analysis. The flowchart of literature screening is shown in Figure 1.

### 2.4 Analysis tool

CiteSpace is a citation visualization analysis software under the background of scientometrics and data visualization (26). CiteSpace can visualize and analyze the literature in WoS, PubMed, CNKI, and other databases to present the structure and distribution of scientific knowledge, help researchers to quickly sort out the history of the development of a certain field, find out the key literature and keywords, and identify the research frontiers and development trends in the field (27, 28). This study utilized CiteSpace V.6.1. R6 (64-bit) to analyze relevant research. Bibliometrix is the R package for bibliometrics that integrates scientific cartographic analysis (29), which can automatically convert and analyze the bibliographic information of the selected publications, including country, institution, journal distribution, year of publication, authors, and keywords. The Bibliometrix package in R 4.4.2 was used to extract the main information, construct the global distribution network, and create a country-author-keywords three-field plot.

### 2.5 Data analysis

CiteSpace was used to analyze publications on country, institution, author, cited journal, cited reference, and keywords. The time slice ranges from January 2005 to December 2024, with an interval of 1 year. CiteSpace can generate a visual knowledge graph consisting of nodes representing different elements, such as institution or country, and links between nodes indicating cooperative or co-cited relationships. The size of the node reflects the frequency of the term, and the larger the node, the higher the frequency. The nodes are shown as circles, and the width of the circle in a given year represents how many terms appear in that year, and the wider the more terms appear. Different colors of the circle indicate different years, darker ones indicate earlier years, and lighter ones indicate more recent years. In addition, the purple outer ring represents centrality, and a thicker purple outer ring indicates greater centrality. Nodes with high centrality are generally considered to be turning points or pivotal points in the field (30). The Journal Impact Factors (IF) cited in this analysis are from the 2024 edition of the Journal Citation Reports (Clarivate Analytics).

## 3 Results

### 3.1 Annual publication analysis

**Figure 2:**
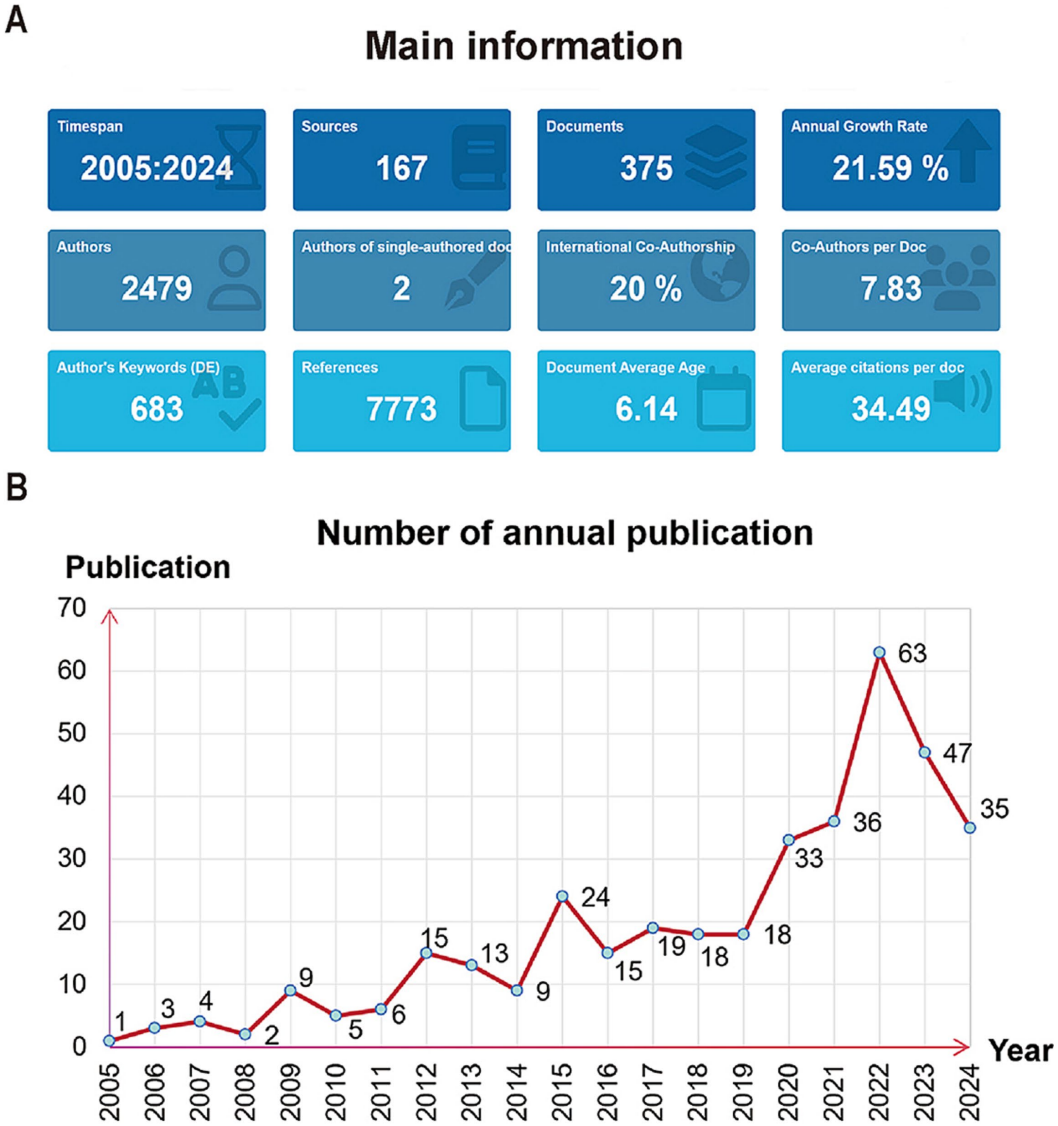
**(A)** The main information of the retrieved publications. **(B)** The number of annual publications from 2005 to 2024.

Through our search strategy, we manually excluded irrelevant literature, and finally, a total of 375 publications were included in this study. The relevant data of retrieved publications (as shown in Figure 2A) included 167 sources, 2,479 authors, and 7,773 references. Furthermore, the document’s average age is 6.14, which indicates that critical rehabilitation is an emerging and promising research field. As can be seen from Figure 2B, the number of published articles in the field of early rehabilitation of patients with MV has shown a steady growth trend in general in the past 20 years, and the annual growth rate is 21.59%, indicating that this field is currently a hot spot of concern for researchers. According to the degree of variation, the annual number of publications can be classified into two distinct stages. The first stage is the slight growth stage: From 2005 to 2015, the total number of published papers during this period was 91, and the maximum difference in the annual number of published papers was only 23. The second stage is the significant growth stage: From 2016 to 2024, the total number of papers published during this period was 284, and the maximum of the annual publication difference was 48. It can be seen that the study of early rehabilitation of patients with MV is attracting increasing attention and may become a research hotspot and focus in the future.

### 3.2 Country and institution analysis

**Figure 3:**
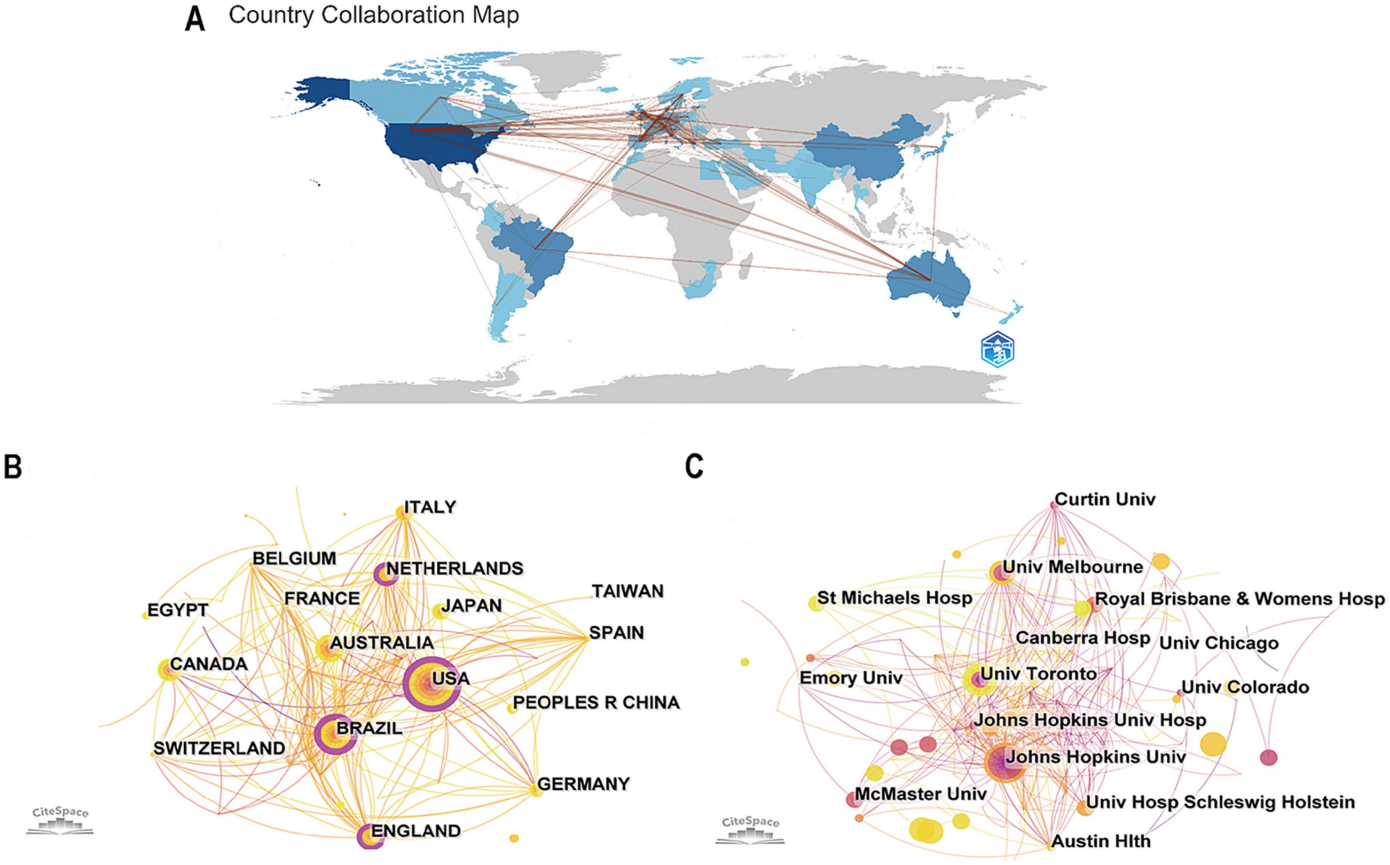
**(A)** The country’s collaboration map. **(B)** The co-occurrence map of countries. **(C)** The co-occurrence map of institutions (the lines connecting represent the mutual cooperation between countries/institutions).

To probe the connection between studies published in each country, we analyzed all articles on early rehabilitation of patients with MV from 2005 to 2024 in 1-year slices. It produced a country co-occurrence map with a merged network of 55 nodes and 202 links shown in Figure 3B. The country with the largest number of publications is the United States (99), followed by Brazil (42), Australia (39), England (28), and China (27). The top-ranked country by centrality is the United States with a centrality of 0.36, followed by Brazil (0.24) and Australia (0.14). The United States demonstrates significantly higher publication volume and centrality compared to other nations, solidifying its position as the cornerstone of this research field with unparalleled influence and academic leadership. Although China, Italy, Canada, and Japan occupy the top ten in the number of annual publications, their centrality is low. And a map of the country’s collaboration is shown in Figure 3A. Different colors represent disparate yields, with darker blue indicating that the country is publishing more articles in the field. The lines between countries represent their cooperation and connection. Research collaborations were primarily concentrated in North America, Europe, Oceania, and East Asia, with particularly strong ties between Europe and the United States but weaker connections between Asia and South America.

Setting “institution” as the node type, we generated a co-occurrence map of institutions with a network of 354 nodes and 634 links as shown in Figure 3C. The institution with the largest number of publications is Johns Hopkins University (17, the United States), then the University of Melbourne (9, Australia), the University of Toronto (8, Canada), and Austin Health (7, Australia). The leading institutions are primarily from the United States and Australia, yet low centrality scores indicate insufficient collaboration. Moreover, China, Japan, Canada, and other nations should focus on in-depth research on early rehabilitation for patients with MV while fostering closer international cooperation with other countries. The top ten most productive countries and institutions in this area of study are shown in Table 1.

**Table 1:** The top ten countries and institutions.

Ranking	Country	Frequency	Centrality	Institution	Frequency	Centrality
1	The United States	99	0.36	Johns Hopkins University	17	0.10
2	Brazil	42	0.24	University of Melbourne	9	0.03
3	Australia	39	0.08	University of Toronto	8	0.06
4	England	28	0.14	Austin Health	7	0.06
5	China	27	0.00	Curtin University	6	0.01
6	Italy	22	0.08	Royal Brisbane and Women’s Hospital	5	0.02
7	Japan	21	0.00	University of Colorado	5	0.01
8	Germany	17	0.09	Canberra Hospital	4	0.01
9	Canada	17	0.07	University Hospital Schleswig-Holstein	4	0.01
10	Netherlands	14	0.13	University of Chicago	4	0.00

### 3.3 Cited journal analysis

**Figure 4:**
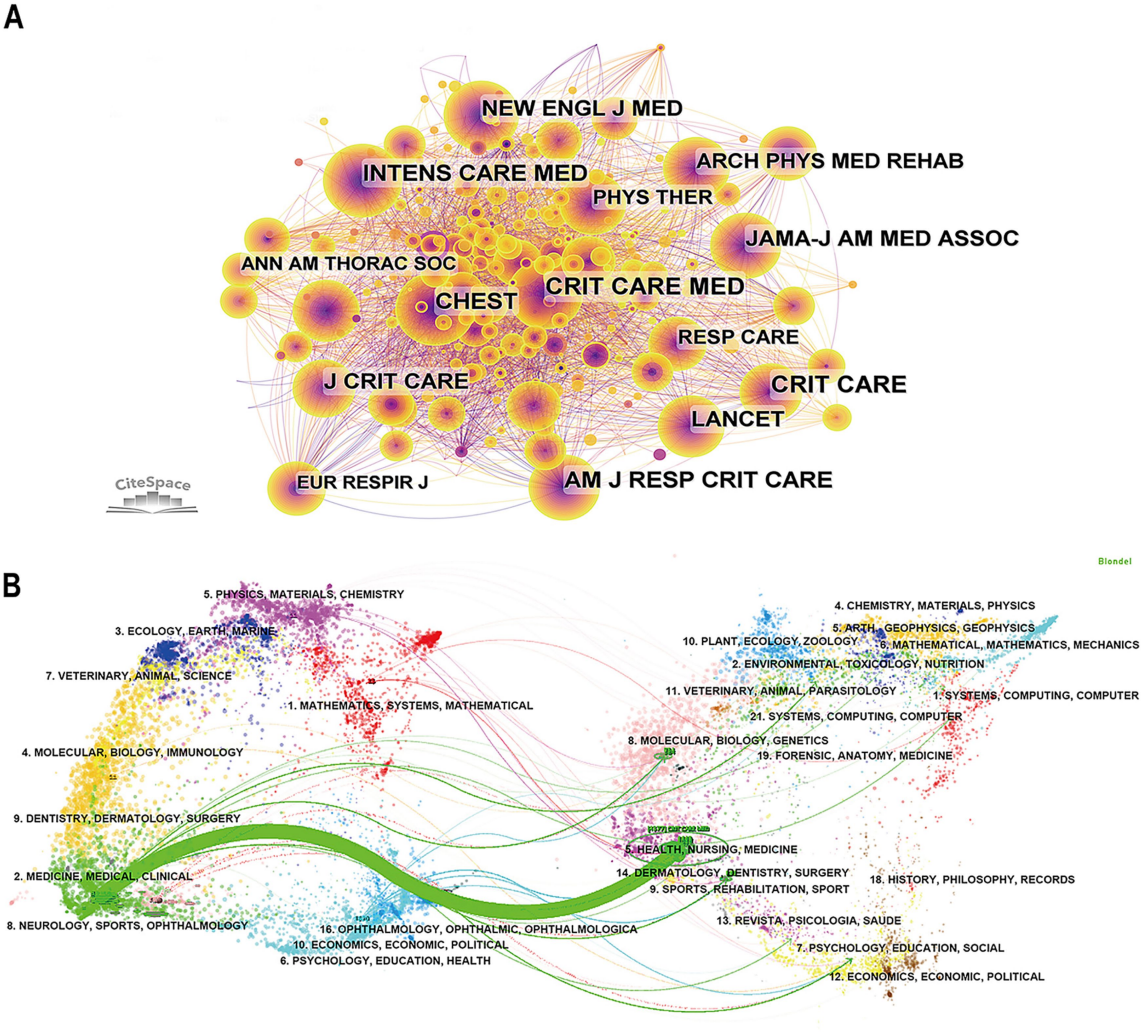
**(A)** The co-occurrence map of cited journals. **(B)** The dual-map overlay of cited journals (nodes of different colors represent various disciplines, the labels on the nodes indicate their categories, and the lines show the connections between disciplines).

Visualizing the analysis with “cited journal” as nodes, all articles on the critical rehabilitation from 2005 to 2024 were sliced with a time interval of 1 year. Then 455 nodes and 3,621 links were obtained for cited journals (Figure 4A). The most cited journal is Critical Care Medicine (292), then is Critical Care (247), Intensive Care Medicine (242), and American Journal of Respiratory and Critical Care (222). The top ten cited journals in this field and their centrality are shown in Table 2. The highest centrality of the cited journal is Anesthesia and Intensive Care (IF = 1.2) with 0.11, followed by American Journal of Critical Care (IF = 2.2) with 0.09 and Anesthesiology (IF = 9.1) with 0.09.

**Table 2:** The top ten cited journals.

Ranking	Cited journal	Frequency	Centrality	IF (2024)
1	Critical Care Medicine	292	0.01	6.0
2	Critical Care	247	0.01	9.3
3	Intensive Care Medicine	242	0.00	21.2
4	American Journal of Respiratory and Critical	222	0.02	19.4
5	Chest	204	0.01	8.6
6	JAMA-Journal of the American Medical Association	189	0.00	55
7	Lancet	182	0.00	88.5
8	Journal of Critical Care	171	0.03	2.9
9	New England Journal of Medicine	159	0.01	78.5
10	Archives of Physical Medicine and Rehabilitation	130	0.03	3.7

The dual-map overlay of the journal can intuitively represent the research dynamics of the discipline (Figure 4B). Each point on the dual-map overlay represents a journal category, which is mainly composed of two parts: the left is the citing journal, the right is the cited journal, and the curve between them is the path of citation (31, 32). We can see that most publications were published in journals related to mathematics, physics, ecology, molecular, medicine, medical, clinical, and neurology. Moreover, most publications were cited in journals related to systems, nutrition, materials physics, health, nursing, rehabilitation, and psychology education. There is one main citation path, the green curve, which indicates that journals in the field of health, nursing, and medicine are predominantly cited by journals in the field of medicine, medical, and clinical.

### 3.4 Author and cited reference analysis

**Figure 5:**
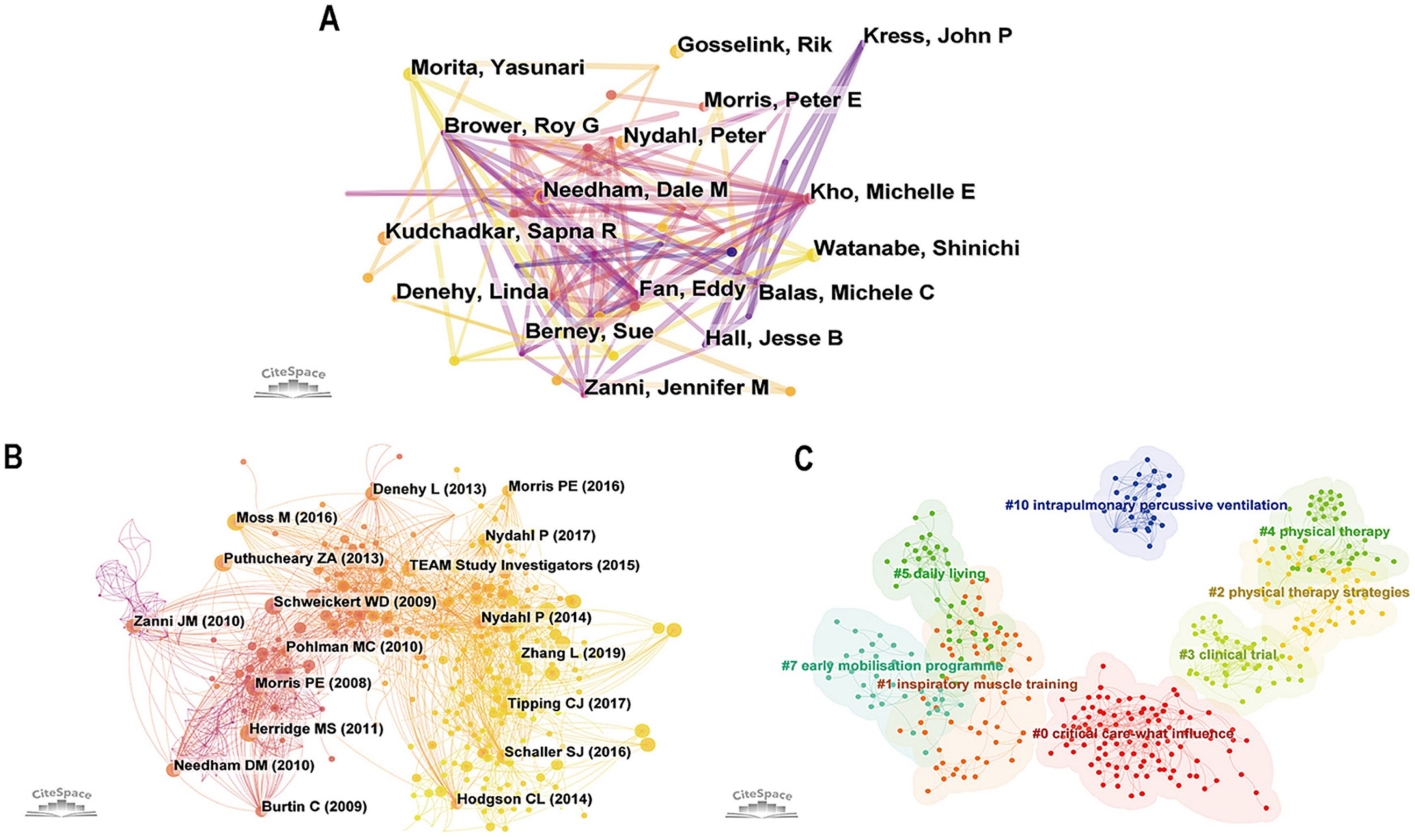
**(A)** The co-occurrence map of authors. **(B)** The co-occurrence map of cited references. **(C)** The cluster map of cited reference (different colors represent different clusters).

Selecting “author” as the node type analysis, we obtained the co-author network map with the combined network of 473 nodes and 807 links, as shown in Figure 5A. The top three active authors are Needham Dale M (9), Kho Michelle E (6), and Denehy Linda (6). Table 3 lists the top ten authors who published critical rehabilitation-related articles and their centrality. They are active and influential authors in this field. The most active academic research group came from America, followed by Australia, Canada, and Belgium.

**Table 3:** The top ten authors.

Ranking	Author	Country	Affiliation	Frequency	Centrality
1	Needham, Dale M	The United States	Johns Hopkins University	9	0.00
2	Kho, Michelle E	Canada	McMaster University	6	0.00
3	Denehy, Linda	Australia	University of Melbourne	6	0.00
4	Zanni, Jennifer M	The United States	Johns Hopkins University	4	0.00
5	Morris, Peter E	The United States	University of Alabama Birmingham	4	0.00
6	Berney, Sue	Australia	Institute for Breathing and Sleep	4	0.00
7	Balas, Michele C	The United States	Ohio State University	3	0.00
8	Gosselink, Rik	Belgium	University of Leuven	3	0.00
9	Nydahl, Peter	Germany	University Hospital of Schleswig-Holstein	3	0.00
10	Fan, Eddy	Canada	University of Toronto	3	0.00

Selecting “reference” as node types, finally generated a co-citation network map with a merged network of 638 nodes and 2,566 links, as shown in Figure 5B. The top ten most cited references in this area of study are shown in Table 4 (17, 33–41). Early physical and occupational therapy in mechanically ventilated, critically ill patients: a randomized controlled trial, written by Schweickert WD in 2009, is the most cited literature. Followed by Early intensive care unit mobility therapy in the treatment of acute respiratory failure by Morris Peter in 2008, and Safety of Patient Mobilization and Rehabilitation in the Intensive Care Unit. Systematic Review with Meta-Analysis written by Nydahl Peter in 2017. It is evident that the majority of co-citations focus on the effects of active mobilization and rehabilitation in the ICU on mortality and functional outcomes, with primary interventions including physical therapy and occupational therapy. The cited references were predominantly published in earlier years, reflecting the enduring influence of classical literature in the field. It may also be related to the time lag required for recent studies to accumulate citations and the current scarcity of high-quality evidence-based research. Therefore, future efforts should focus on exploring new research hotspots and conducting more high-level studies to advance the field.

**Table 4:** The top ten cited references.

Ranking	Title	Year	Frequency	Centrality	References
1	Early physical and occupational therapy in mechanically ventilated, critically ill patients: a randomized controlled trial	2009	26	0.02	(13)
2	Early intensive care unit mobility therapy in the treatment of acute respiratory failure	2008	23	0.03	(29)
3	Safety of patient mobilization and rehabilitation in the intensive care unit. systematic review with meta-analysis	2017	21	0.04	(30)
4	Functional disability 5 years after acute respiratory distress syndrome	2011	21	0.10	(31)
5	Early physical medicine and rehabilitation for patients with acute respiratory failure: a quality improvement project	2010	20	0.14	(32)
6	A randomized trial of an intensive physical therapy program for patients with acute respiratory failure	2016	20	0.15	(33)
7	Early mobilization of critically ill patients in the intensive care unit: a systematic review and meta-analysis	2019	19	0.10	(34)
8	Early mobilization and recovery in mechanically ventilated patients in the ICU: a bi-national, multi-center, prospective cohort study	35	19	0.02	(35)
9	Early, goal-directed mobilization in the surgical intensive care unit: a randomized controlled trial	2016	18	0.08	(36)
10	Acute skeletal muscle wasting in critical illness	2013	18	0.04	(37)

Based on co-occurrence analysis, the log-likelihood ratio is used to cluster references, and a reference clustering network diagram was created (Figure 5C). A clustering modularity value (Q) greater than 0.3 indicates the effectiveness of the clustering, while a clustering silhouette index (S) greater than 0.7 validates the reliability of the cluster analysis results (42). The cited reference cluster exhibits a Q value of 0.7712 and an S value of 0.8904, indicating high effectiveness and reliability. A total of 8 clusters were obtained, including #0 critical care-what influence, #1 inspiratory muscle training, #2 physical therapy strategies, #3 clinical trial, #4 physical therapy, #5 daily living, #7 early mobilization program, #10 intrapulmonary percussive ventilation. Among the clusters, #1, #2, #4, #7, and #10 mainly focus on rehabilitation interventions for patients with MV in the ICU. Clusters #1 is about the outcomes and impacts of ICU patients, while clusters #3 is primarily concerned with study designs and methodological approaches. The most impactful studies in this field are predominantly centered on physical therapy and pulmonary rehabilitation. The specific information of the references cluster labels is shown in Table 5.

**Table 5:** The reference cluster label lists.

Cluster-ID	Cluster name	Size	Silhouette	Cluster label
#0	Critical care-what influence	102	0.777	Of-bed rehabilitation; critical care-what influence; post-intensive care; ill patient; acute respiratory failure; speech therapy; hospital-specific factor
#1	Inspiratory muscle training	60	0.875	Inspiratory muscle training; case report; observational study; acute rehabilitation; covid-19 disease; subacute rehabilitation
#2	Physical therapy strategies	47	0.881	Ill patient; physical therapy; unit-acquired weakness; neuromyopathy; physical therapy strategies; acute respiratory failure; pulmonary rehabilitation; rehabilitation therapy; interactive video game
#3	Clinical trial	46	0.921	Intensive care unit; clinical trial; abcde bundle; post-intensive care unit; randomized clinical trial; chest muscle; enhancing rehabilitation
#4	Physical therapy	38	0.920	Physical therapy; intensive care; unit-acquired weakness; critical illness neuromyopathy; ill patient; case series; patient outcome; following icu-acquired weakness; physical therapy management; acute lung injury
#5	Daily living	36	0.914	Rehabilitation intensity; early nutrition; extremity strength; observational study; critical care cycling; health-related quality; prolonged intensive
#7	Early mobilization program	33	0.923	Early mobilization program; short-term outcome; intensive care unit; novel coronavirus patient; lower-extremity muscle weakness; controlled trial; muscle strength
#10	Intrapulmonary percussive ventilation	27	0.985	Intrapulmonary percussive ventilation; tracheostomized patient; randomized controlled trial; mechanical ventilation; arm training

### 3.5 Keyword analysis

**Figure 6:**
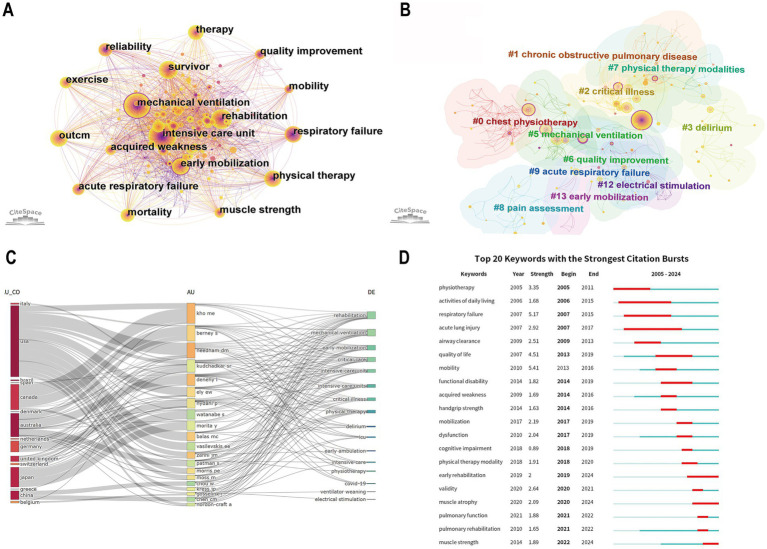
**(A)** The co-occurrence map of keywords. **(B)** The cluster map of keywords. **(C)** Three-field plot showing the network between countries (left), authors (middle), and keywords (right). **(D)** Visualization of the top 20 keywords with the strongest citation bursts.

“Keywords” were selected as node types, and a combined network of keyword co-occurrence graphs with 399 nodes and 2,614 links was generated (Figure 6A). The top 10 high-frequency keywords are intensive care unit (163), mechanical ventilation (149), rehabilitation (80), early mobilization (56), outcome (51), physical therapy (42), therapy (41), survivor (39), acute respiratory failure (32), and exercise (26). Based on co-occurrence analysis, the keyword clustering network diagram was generated, with a Q value of 0.708 and an S value of 0.88 (Figure 6B). Ten clusters were produced including chest physiotherapy (#0), critical illness (#2), delirium (#3), mechanical ventilation (#5), quality improvement (#6), physical therapy modalities (#7), pain assessment (#8), acute respiratory failure (#9), electrical stimulation (#12), and early mobilization (#13). The specific information of keyword cluster labels is shown in Table 6.

**Table 6:** The keyword cluster label lists.

Cluster-ID	Cluster name	Size	Silhouette	Cluster label
#0	Chest physiotherapy	50	0.891	Mechanical ventilation; acute respiratory failure; in-bed cycling; passive joint movement; respiratory mechanics
#2	Critical illness	36	0.857	Critical illness; respiratory therapy; cycle ergometry; mechanical ventilation; resistance training; endurance training; functional outcome; functional training
#3	Delirium	33	0.874	Intensive care unit; controlled trial; safety assessment; rehabilitation; mechanical ventilation; post-intensive care syndrome; intubated patient; risk factor; adverse event
#5	Mechanical ventilation	29	0.908	Mechanical ventilation; physical therapy; hemodynamic monitoring; subject-ventilator asynchrony
#6	Quality improvement	27	0.819	Intensive care; physical strength examination; electrical stimulation; rehabilitation nursing; interactive video games
#7	Physical therapy modalities	26	0.816	Acute respiratory distress syndrome; pulmonary rehabilitation; early bedside cycle exercise; mechanical insufflation-exsufflation
#8	Pain assessment	24	0.914	Occupational therapy; long-term quality; stress disorders; barthel index score; intensive care unit; mechanical ventilators; long-term quality
#9	Acute respiratory failure	15	0.901	Mechanical ventilation; critical illness; physical function; acute lung injury; controlled trial
#12	Electrical stimulation	27	0.951	Electrical stimulation; ventilator weaning; deep brain stimulation; emergency neurosurgery; muscle weakness; physical function
#13	Early mobilization	15	0.895	Medical complications; vascular access device; neurocritical care; brain injury; body weight support; mobility

To better understand the relationship among authors, countries, and keywords, a three-field plot was generated (Figure 6C). The width of the connecting lines between nodes is proportional to the strength of connections, and the wider the lines, the more connections there are. We can see that the United States (flow count is 17) was the most connected country, followed by Australia (flow count is 14), Germany (flow count is 6), and Canada (flow count is 5). The scholars who contributed the most keywords were Berney Sue and Denehy, Linda from Australia, and Needham Dale M from the United States. This finding further underscores the significant contributions of the United States and Australia to advancements in this field.

“Burst” means a sudden increase over a period of time. The burst word analysis can help detect the burst word with a high frequency change rate and rapid growth rate, and then analyze the frontier field and development trend of this discipline (43). The top 20 keywords with the strongest citation bursts from 2005 to 2024 are shown in Figure 6D. The blue line indicates the time intervals, and the red line indicates the time of the keyword outbreak. In the field of early rehabilitation of patients with MV research, physiotherapy, activities of daily living, respiratory failure, and airway clearance are earlier keywords (before 2010), while muscle atrophy, pulmonary rehabilitation, and muscle strength are the most recent keywords to appear (after 2020). In addition, mobility, respiratory failure, quality of life, and physiotherapy have a large strength. Pulmonary rehabilitation, cognitive training, and muscle strength training may be the future trend of early rehabilitation of patients with MV. The evolution of keywords highlights the advances in the field in optimizing patient outcomes and quality of life, reflecting the gradual maturation of early rehabilitation practices.

**Figure 7:**
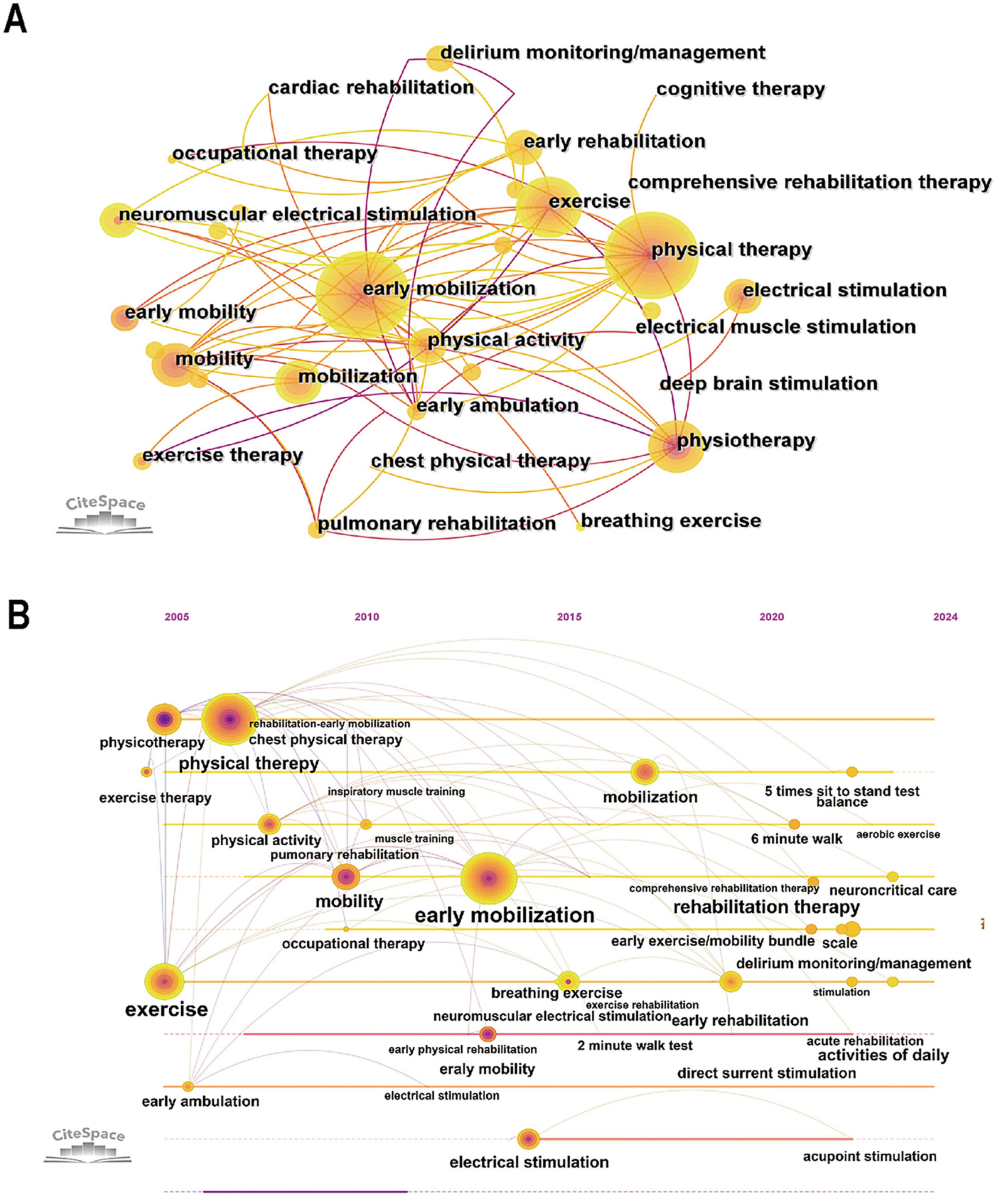
**(A)** The co-occurrence map of rehabilitation treatment-related keywords. **(B)** The timeline map of rehabilitation treatment-related keywords.

To further investigate research hotspots and emerging trends in rehabilitation interventions within this field, we systematically identified and extracted rehabilitation treatment-related keywords for visual analysis (Figure 7A). The main rehabilitation treatments included early mobilization, physical therapy, exercise, and mobility. Additionally, electrical stimulation, occupational therapy, and pulmonary rehabilitation are also utilized. In contrast, cardiac rehabilitation, cognitive therapy, and chest physical therapy are frequently applied. The timeline (Figure 7B) illustrates the evolution of keywords over the past two decades and forecasts their future trends. Early rehabilitation interventions primarily focus on exercise, physical therapy, and early ambulation. Between 2010 and 2015, occupational therapy, inspiratory muscle training, breathing exercises, and electrical stimulation began to gain attention. More recently, acupoint stimulation, delirium monitoring/management, and comprehensive rehabilitation therapy have started to receive increasing attention.

## 4 Discussion

### 4.1 Research status

This bibliometric analysis probed the development of research related to early rehabilitation of patients with MV in the past 20 years via CiteSpace and Bibliometrix. Over the past few years, particularly since 2019, there has been a significant upward trend in the number of publications, which peaked in 2022. This surge is likely closely associated with the coronavirus disease 2019 (COVID-19). COVID-19 affects the respiratory system, skeletal muscles, nervous system, and nearly all bodily systems, exacerbating lung injury, muscle fatigue, and dyspnea. Furthermore, it prolongs ICU stays, significantly reduces mechanical efficiency, and extends the duration of MV (44, 45). The global COVID-19 pandemic led to a surge in critically ill patients, which directly underscored and validated the necessity and safety of early rehabilitation in the ICU, thereby driving a rapid expansion in clinical practice and research within this field. The United States, Brazil, Australia, and England are major contributing countries. North American and European countries have established the most intensive cooperation networks. Most active scholars, institutions, and journals in this field are from the United States, Canada, Australia, and England. This trend is likely attributed to the robust policy support and substantial financial investment in healthcare within these countries (46–48). Additionally, leading universities and research institutions in North America and Europe have significantly strengthened collaboration networks.

Current communication and collaboration in this field are predominantly confined to the domains of clinical medicine and related medical disciplines. However, given the complexity of providing early rehabilitation to ICU patients with MV, future studies would benefit significantly from interdisciplinary approaches. For instance, integrating materials science could advance the development of more sophisticated rehabilitation equipment, while insights from ecological research could contribute to more sustainable healthcare practices. Such interdisciplinary collaboration is not only beneficial but essential for driving pivotal advancements in this field.

### 4.2 Research focus and hotspots

According to the cited references and keyword analysis, it can be found that the focus of current research on early rehabilitation of patients with MV is the major complications and their management in the ICU, the safety and efficacy of early rehabilitation in patients with MV, and the physical therapy strategies in patients with MV. Fan E believes that further research is needed to elucidate the mechanisms leading to neuromuscular dysfunction and injury, and to better understand the relationship between ICU-acquired weakness, physical function, and quality of life in patients (49). Additionally, studies have shown that early mobilization and rehabilitation can reduce the incidence of ICU-acquired weakness, with less likelihood of adverse events, and are safe and feasible in the ICU (50). And the risk of adverse outcomes associated with early rehabilitation is minimal, with a meta-analysis reporting an overall cumulative incidence of 2.6% for potential safety events and only 0.6% for rare medical complications (34).

Although physical therapy has been widely advocated to improve dysfunction in patients with MV, clinical trials have reported different effects on the outcomes of physical therapy interventions. González-Seguel F et al. thought that this can be explained by a lack of awareness of the optimal dosage of physical therapy for a patient (e.g., frequency, intensity) or that a mismatch between the ventilatory support and exercise-induced ventilatory demand during physical therapy may overly increase the work of breathing, leading to exhaustion, which in turn limits the effectiveness of physiotherapy (51). Therefore, the setting and monitoring of physical therapy in patients with MV is a significant focus and trend in future research. Additionally, different professional groups may influence clinical decisions due to different perceptions, prejudices, and role perceptions (52). Future studies should be conducted to understand when to apply rehabilitation therapy, which treatment is more effective, and the synergistic relationship with other care measures, rather than relying on subjective biases and different perceptions.

Furthermore, our analysis found that respiratory failure emerged as a predominant keyword, inspiratory muscle training was a notably cited reference cluster, and pulmonary rehabilitation was an emerging keyword burst in this field. The results highlight the key role of respiratory management and pulmonary rehabilitation in patients with MV. As prolonged MV leads to diaphragmatic atrophy, pulmonary rehabilitation such as respiratory muscle training, deep breathing exercises, induced spirometry, postural drainage, and sputum expectoration can reduce ventilation, clear intrapulmonary inflammation, and activate the respiratory muscles (53). Neuromuscular electrical stimulation can improve patients’ functional independence and shorten MV time, but it is applied to peripheral muscles in a higher proportion and to respiratory muscles and their auxiliary muscles less (54). More studies are needed to evaluate its actual effectiveness in order to better promote this intervention. Huang et al. found that most pulmonary rehabilitation items were bundled, without explaining the individual role of each item. Intervention plans for pulmonary rehabilitation and MV should be further clarified, based on standardized treatment and long-term observation (55). In addition, future pulmonary rehabilitation can utilize new technologies such as virtual reality, brain-computer interface, and artificial intelligence to enhance the amusement and effectiveness of rehabilitation training, thereby improving patient participation and compliance. Simultaneously, it is essential to explore the unique therapeutic approaches of Traditional Chinese Medicine in pulmonary rehabilitation. By integrating Traditional Chinese Medicine pulmonary rehabilitation with modern medical rehabilitation methods, a distinctive pulmonary rehabilitation model with Chinese characteristics can be established, which will significantly enhance China’s influence in this field.

In addition to ICU-acquired weakness and pulmonary dysfunction, which are the two major challenges in early ICU rehabilitation mentioned above, delirium and disorder of consciousness (DoC) are also crucial issues requiring urgent attention. Delirium (keyword cluster #3), characterized by acute cognitive and attentional disturbances, and DoC (keyword burst: cognitive impairment), featured by arousal and awareness alterations, are highly prevalent in ICU patients with MV and linked to prolonged ventilation, extended hospital stays, functional impairment, and increased caregiver burden (56, 57). Statistical data indicate that the prevalence of delirium is generally 50 to 70% in MV patients (58). However, rehabilitation treatment-related analysis reveals a persistent focus on physical therapy and early mobilization, while cognitive therapy remains underutilized. The timeline of rehabilitation intervention-related keywords shows that delirium monitoring/management is an emerging keyword in recent years, highlighting cognitive rehabilitation as a focus and trend in future research. A meta-analysis found that multi-sensory stimulation, telling structured stories with a familiar voice, and transcranial direct current stimulation positively contribute to the recovery from DoC (59). Acupuncture has also been demonstrated to effectively improve consciousness levels, with the advantages of being well-tolerated, reversible, and having rare adverse events (60). In addition, transcutaneous vagus nerve stimulation, an important extension of acupuncture therapy, has been shown to improve DoC (61). Therefore, the application of acupuncture in the ICU to promote patient awakening holds significant potential for future research. In conclusion, cognitive rehabilitation has emerged as a burgeoning research trend, with the potential to fill a significant gap in this field. Future studies should be dedicated to devising and validating more novel cognitive interventions to improve prognoses for MV patients with delirium and DoC.

### 4.3 Study strengths and limitations

To our knowledge, this is the first bibliometric analysis on the rehabilitation of patients with MV in the ICU. This is a prospective study, the literature search terms are specific and comprehensive, and the results are clearly and intuitively presented.

This study also had some limitations. First, in this study, only studies published in the WoSCC were included, and the paper types were “article” in English, which may have neglected other high-quality literature in the field, resulting in some limitations in the literature search. Second, due to the presence of synonyms, there may be some overlap when analyzing the co-occurrence and clustering of keywords. Furthermore, bibliometric methodology cannot effectively take into account the scientific robustness or validity of a publication. Highly cited publications do not necessarily have high scientific quality and do not necessarily reflect the research hotspots comprehensively. As noted, this analysis did not systematically include pediatric studies. Early rehabilitation for mechanically ventilated children is equally crucial and safe, but must account for distinct developmental and physiological characteristics (62, 63). The link between normal baseline function and delayed rehabilitation in critically ill children highlights the necessity and urgency of early intervention (64). Unlike adult rehabilitation, pediatric care requires adjusted respiratory support and development-matched play-based strategies (65). Future research should conduct in-depth analyses of early rehabilitation for pediatric ICU patients with MV.

## 5 Conclusion

For the past few years, research related to early rehabilitation of patients with MV in the ICU has received increasing attention, and the growing number of publications indicates the growing importance of this research area. Through visual analysis of countries and institutions, cited journals and references, authors and keywords, etc., we found that the United States is a highly prolific and influential country, the communication and cooperation between various countries and authors is close, and the intervention, management, and assessment of early rehabilitation of patients with MV are current research hotspots. In summary, this study provides a valuable and forward-looking perspective on early rehabilitation of patients with MV in the ICU research, which helps to understand the main research countries and institutions, high-impact journals, and global research hotspots and development trends.

## Data Availability

Publicly available datasets were analyzed in this study. This data can be found on the Web of Science Core Collection, further inquiries can be directed to the corresponding author.
